# Timing of HIV Seroreversion Among HIV-Exposed, Breastfed Infants in Malawi: Type of HIV Rapid Test Matters

**DOI:** 10.1007/s10995-016-2152-4

**Published:** 2016-09-08

**Authors:** Emily R. Smith, Michael Hudgens, Anna D. Sheahan, William C. Miller, Stephanie Wheeler, Julie A. E. Nelson, Queen Dube, Annelies Van Rie

**Affiliations:** 1 Department of Epidemiology, Gillings School of Global Public Health, University of North Carolina, Chapel Hill, NC USA; 2 Department of Biostatistics, Gillings School of Global Public Health, University of North Carolina, Chapel Hill, NC USA; 3 Division of Infectious Diseases, Department of Medicine, University of North Carolina at Chapel Hill, Chapel Hill, NC USA; 4 Department of Health Policy and Management, Gillings Schools of Global Public Health, University of North Carolina, Chapel Hill, NC USA; 5 Department of Microbiology and Immunology, School of Medicine, University of North Carolina, Chapel Hill, NC USA; 6 Malawi-Liverpool-Wellcome Trust Clinical Research Programme, Queen Elizabeth Central Hospital, Blantyre, Malawi; 7 Epidemiology and Social Medicine, University of Antwerp, Antwerp, Belgium

**Keywords:** Breastfeeding, Diagnostic testing, Early infant diagnosis, HIV transmission, Prevention of mother-to-child transmission

## Abstract

*Introduction* Rapid HIV serological tests are a cost-effective, point-of-care test among HIV exposed infants but cannot distinguish between maternal and infant antibodies. The lack of data on the timing of decay of maternal antibodies in young infants hinders the potential use of rapid tests in exposed infants. We aimed to determine the time to seroreversion for two commonly used rapid tests in a prospective cohort of HIV-exposed breastfeeding infants ages 3–18 months of life. *Methods* We collected data on the performance of two commonly used rapid tests (Determine and Unigold) in Malawi between 2008 and 2012 or at the University of North Carolina between 2014 and 2015. Time to seroreversion was estimated for both rapid tests using the Kaplan–Meier product limit estimator which allows for interval censored data. *Results* At 3 months of age, 3 % of infants had seroreverted according to Determine and 7 % had seroreverted according to Unigold. About one in four infants had achieved seroreversion by 4 months using Unigold, but only about one in twelve infants by 4 months when using Determine. More than 95 % of all infants had seroverted by 7 months according to Unigold and by 12 months according to the Determine assay. *Discussion* We show that the time of seroreversion depends greatly on the type of test used. Our results highlight the need for recommendations to specify the timing and type of test used in the context of infant HIV detection in resource-poor settings, and base the interpretation of test result on knowledge of time to seroreversion of the selected test.

## Significance

The need for an accurate rapid, point-of-care HIV test for breastfeeding infants in resource-limited settings is now greater than ever, given the recommendation for prolonged breastfeeding and increasing access to life-saving treatment for HIV-infected infants. However, circulating maternal HIV antibodies complicates the interpretation of rapid serological tests in HIV exposed, uninfected infants. The lack of data on the timing of decay of maternal antibodies in young infants hinders the potential use of rapid tests in exposed infants. We show that the time of seroreversion depends greatly on the type of test used.

## Introduction

The need for an accurate rapid, point-of-care HIV test for breastfeeding infants in resource-limited settings is now greater than ever, given the recommendation for prolonged breastfeeding and increasing access to life-saving treatment for HIV-infected infants. Since 2010, the World Health Organization (WHO) breastfeeding guidelines (WHO 2010) encourage women to exclusively breastfeed for the first six months of life and continue breastfeeding throughout the first two years of life, whereas previously, HIV positive mothers were encouraged to use formula feeding starting at age 6 months (WHO 2006). In light of the continued risk of vertical HIV infection that accompanies such prolonged breastfeeding, consensus statements have concluded that a prompt and definitive HIV diagnosis among infants is crucial for timely initiation of life-saving antiretroviral treatment (Chiappini et al. 2006; Prendergast et al. 2008; Violari et al. 2008).

For early infant diagnosis (EID) of HIV infection, the WHO recommends a virological assay. Use of virological assays in resource-limited settings is limited by high costs and logistical constraints, as these assays require transportation to a centralized laboratory and thus a return visit by the mothers for test results. Rapid serological tests could be a cheaper, point-of-care alternative. The lack of data on the timing of decay of maternal antibodies in young infants hinders the potential use of rapid tests for identification of new HIV infections in breastfeeding infants. Furthermore, circulating maternal HIV antibodies in HIV exposed, uninfected infants complicates the interpretation of rapid serological tests due to the difficulty in distinguishing between HIV antibodies produced by an HIV-infected infant and maternal HIV antibodies in an HIV exposed, uninfected infant (Moodley et al. 1995; Sirinavin and Atamasirikul 2000).

We aimed to determine the time to seroreversion for two commonly used rapid tests in a cohort of HIV-exposed breastfeeding infants ages 3–18 months of life.

## Methods

Between May 2008 and March 2012, we prospectively collected data on two HIV rapid tests, Unigold^®^ and Determine^®^ in two healthcare centers of the Blantyre region of Malawi as part of a community-based cohort study examining the effect of HIV infection and HIV exposure on neurodevelopment. During that period, both clinics provided HIV counseling and testing to all pregnant women and offered single dose nevirapine or zidovudine treatment to women who were HIV infected and to infants born to HIV infected mothers. Option B+ was not implemented throughout the country during this time period. Children were included if they were HIV negative by HIV DNA PCR (version 1.5 of the Amplicor HIV-1 DNA, Roche, Basel, Switzerland) at age 6 weeks or the age closest to that time (median age of enrollment was 6.4 weeks, standard deviation 2.6 weeks). Caregivers were asked to bring these infants to the study clinic at age 10 weeks and every 3 months between age 14 weeks and 18 months. At all timepoints, dried blood spots were collected and stored for future HIV DNA PCR assays, and a venous whole blood sample was taken and stored at ≤−70 °C. A positive HIV DNA PCR result was confirmed through a second HIV DNA PCR assay or an HIV RNA viral load assay. HIV antibody testing was not conducted after a confirmed positive HIV DNA PCR result. Starting in February 2009, an additional fingerprick was performed for point-of-care Unigold^®^ and Determine^®^ rapid HIV tests at age 6, 9, 12, 15, and 18 months in real-time during the study visit. To obtain data at age 3 months, the Unigold^®^ and Determine^®^ rapid tests were performed on stored blood. Missing data at other time points, due to failure to perform the rapid test at point-of-care or assay stock-outs, were also resolved by performing rapid HIV tests on stored blood. Stored samples were allowed to thaw per manufacturers instructions, and both rapid tests were performed simultaneously by a single operator at a research laboratory at the University of North Carolina. Weakly positive rapid tests results were reported as positive. The proportion of samples evaluated on stored blood at UNC was 98 % at 3 months, 33 % at 6 months, 32 % at 9 months, 41 % at 12 months, 25 % at 15 months, and 14 % at 18 months. HIV infection status of all infants was confirmed or ruled out with an HIV DNA PCR test at each timepoint.

The primary endpoint, time to seroreversion, was defined as the first occurrence of a negative HIV rapid test, confirmed by a negative HIV PCR test. Because rapid tests were performed at 3-month intervals, the exact time of seroreversion was not observed but only known to have occurred in the time interval between the last positive rapid test and the first negative rapid test. The distribution of age of seroreversion was estimated for both Determine and Unigold using the extension of the Kaplan–Meier product limit estimator which allows for interval censored data via SAS PROC ICLIFETEST (Peto 1973; Turnbull 1976; Wellner and Zhan 1997) (SAS Institute). Observations were right censored at the first occurrence of an incident HIV infection, death, or loss to follow-up, if these occurred prior to seroreversion, or at the end of the 18 month follow-up period. Bootstrap sampling was used to calculate corresponding 95 % confidence intervals. We accounted for competing risks (Aalen and Johansen 1978) of death or HIV infection in the infant. Since these results were similar to the results without accounting for competing risks, all results presented are unaccounted for competing risks (Satagopan et al. 2004).

The University of Malawi College of Medicine Research and Ethics Committee and the University of North Carolina at Chapel Hill Institutional Review Board approved the study protocol. All mothers provided written informed consent and permission for participation of their infant.

## Results

Of the 121 infants included in the analysis, 51 % were female and 49 % were male. The median age of the mother at the time of delivery was 27 years. Most women self-reported no PMTCT for themselves (71 %), and 80 % of infants receiving single dose nevirapine or zidovudine the regimens in use and available during the study period. Approximately 54 % of infants were exclusively breastfed at month 3 and only 18 % were exclusively breastfed at month 6. Any breastfeeding, including mixed feeding, progressively declined with 87 % of infants breastfeeding at 3 months, 69 % at 6 months, 45 % at 9 months, 39 % at 12 months, 26 % at 15 months, and 17 % at 18 months of age. During follow-up, 21 (17 %) infants were diagnosed with incident HIV infection.

The estimated probability of seroreversion between age 3 and 18 months, stratified by rapid test, is presented in Table 1. At 3 months of age, 3 % of infants had seroreverted according to Determine and 7 % had seroreverted according to Unigold. About one in four infants had achieved seroreversion by 4 months using Unigold, but only 8 % by 4 months when using Determine. More than 95 % of all infants had seroverted by 7 months according to Unigold and by 12 months according to the Determine assay.

**Table 1:** Estimated probability of seroreversion at a certain age, by rapid test

Age (months)	Determine probability (95 % CI)	Unigold probability (95 % CI)
3	3.1 (0.0, 6.7)	7.4 (1.0, 13.7)
4	8.1 (1.0, 14.5)	23.9 (14.5, 33.4)
5	16.5 (9.1, 23.9)	33.3 (30.0, 36.4)
6	24.9 (17.6, 32.3)	67.3 (61.7, 72.9)
7	72.4 (71.3, 73.4)	98.9 (96.9, 100.0)
8	72.4 (71.3, 73.4)	99.2 (97.1, 100.0)
9	85.5 (82.3, 88.7)	99.3 (97.3, 100.0)
10	90.4 (87.3, 93.6)	99.4 (97.4, 100.0)
11	92.3 (89.4, 95.1)	99.5 (97.6 (100.0)
12	95.1 (92.2, 97.9)	99.7 (97.7, 100.0)
13	100.0 (95.1, 100.0)	99.8 (97.8, 100.0)

*CI* confidence interval

## Discussion

Circulating maternal HIV antibodies complicates the interpretation of rapid serological tests in HIV exposed, uninfected infants. In this cohort of HIV-exposed infants, we show that the time of seroreversion depends greatly on the type of test used, with time of seroreversion occurring at a much younger age for the Unigold assay compared to the Determine assay.

The WHO EID guidelines only recommend rapid tests for diagnostic purposes starting at age 18 months. Our data suggest that the Unigold assay could be used from month 7 onwards to exclude infant HIV infection in HIV-exposed, breastfeeding infants, as maternal antibodies could no longer be detected by the Unigold assay among 7-month old infants. From a mother’s perspective, an HIV negative diagnosis in her infant is emotionally important and could motivate her to adhere to her antiretroviral therapy during the remaining breastfeeding period. For healthcare providers, documentation of seroreversion would increase confidence in the interpretation of HIV screening by rapid tests during the subsequent months of breastfeeding, as a positive rapid test after documented seroreversion will strongly suggest incident infection demanding further investigation and a negative test after a prior negative test strongly suggests that the child has remained free of HIV.

While data are limited, data on time to seroreversion greatly differ between different rapid tests and across studies of the same rapid test. In industrialized countries using Western Blot assays, median ages at seroreversion were 7–12 months (Andiman et al. 1960; Arico et al. 1991; Chantry et al. 1995). South African infants had documented seroreversion in 98 % for Unigold and 50 % for Determine at 12 months (Sherman et al. 2008), with the Determine results lower than the 95 % for Determine we observed at age 12 months. In another cross-sectional South African study, the proportion of HIV exposed uninfected infants (n = 11) who seroreverted according to Determine was 2.6 % between ages of 2–4 months of age, 13 % between ages of 4–6 months, and 82 % between the ages of 8–10 months of age (Sherman et al. 2012), with the latter percentage higher than the 72 % observed in our cohort. Reasons for differences include usage of whole blood versus serum and small sample sizes between studies and differences in postmenstrual age at delivery, maternal HIV immunoglobulin G (IgG) concentrations, maternal HIV severity, and maternal viral load and CD4 count, as these factors are strong determinants of the amount of placental immunoglobulin G (IgG) transfer (Saji et al. 1999; Malek et al. 1989; Palmeira et al. 2012; van den Berg et al. 2011; Farquhar et al. 1999; Scott et al. 2005).

Several limitations to our study need to be mentioned. First, seroreversion rates found in our study may have been inflated due to testing on stored blood (Sherman et al. 2008; Sherman et al. 2012; Pavie et al. 2010), as higher sensitivities have been documented when rapid tests were performed on stored blood as compared to fresh serum (Sherman et al. 2012; Saji et al. 1999). However, we do not expect the use of stored blood to impact the results substantially as 60 % of all testing was conducted in Malawi in real-time. Second, many countries are moving towards provision of HAART for all HIV positive women (under Option B+) and studies have suggested longer times to seroreversion in the presence of HAART (Gutierrez et al. 2012).

In conclusion, our results highlight the need for recommendations to specify the timing and type of test used in the context of infant HIV detection in resource-poor settings, and base the interpretation of test result on knowledge of time to seroreversion of the selected test. Further research is needed in different resource-poor settings to estimate the time of seroreversion, identify the optimal rapid test for repeat screening of breastfeeding infants, and better understand how determinants of seroreversion in HIV exposed infants may differ between settings.
